# Seed-induced Aβ deposits in the corpus callosum disrupt white matter integrity in a mouse model of Alzheimer’s disease

**DOI:** 10.3389/fncel.2022.862918

**Published:** 2022-08-08

**Authors:** Vanessa Aires, Stephanie Ziegler-Waldkirch, Marina Friesen, Wilfried Reichardt, Daniel Erny, Desiree Loreth, Andrew Harborne, Oliver Kretz, Dominik von Elverfeldt, Melanie Meyer-Luehmann

**Affiliations:** ^1^Department of Neurology, Medical Center – University of Freiburg, Freiburg, Germany; ^2^Faculty of Medicine, University of Freiburg, Freiburg, Germany; ^3^Faculty of Biology, University of Freiburg, Freiburg, Germany; ^4^Department of Radiology, Medical Physics, Medical Center – University of Freiburg, Freiburg, Germany; ^5^German Consortium for Translational Cancer Research (DKTK), Heidelberg, Germany; ^6^German Cancer Research Center (DKFZ), Heidelberg, Germany; ^7^Institute of Neuropathology, University of Freiburg, Freiburg, Germany; ^8^Berta-Ottenstein-Programme, Faculty of Medicine, University of Freiburg, Freiburg, Germany; ^9^Institute of Cellular and Integrative Physiology, University Medical Center Hamburg-Eppendorf, Hamburg, Germany; ^10^Department of Internal Medicine III, University Medical Center Hamburg-Eppendorf, Hamburg, Germany; ^11^Center for Basics in NeuroModulation (NeuroModulBasics), Faculty of Medicine, University of Freiburg, Freiburg, Germany

**Keywords:** Alzheimer’s disease (AD), Aβ, seeding, corpus callosum, oligodendrocytes, glial cells, myelination

## Abstract

Neuropathologically, Alzheimer’s disease (AD) is characterized by the accumulation of amyloid-beta peptide (Aβ) and subsequent formation of the so-called Aβ plaques. Along with neuronal loss, previous studies report white matter anomalies and corpus callosum (CC) atrophy in AD patients. Notably, perturbations in the white matter can be observed years before expected disease onset, suggesting that early stages of disease progression play a role in AD-associated loss of myelin integrity. Through seed-induced deposition of Aβ, we are able to examine alterations of central nervous system (CNS) integrity during the initial stages of plaque formation. In this study, we investigate the impact of Aβ seeding in the CC utilizing various imaging techniques as well as quantitative gene expression analysis and demonstrate that Aβ deposits result in an imbalance of glial cells in the CC. We found increased amounts of phagocytic microglia and reactive astrocytes, while oligodendrocyte progenitor cell (OPC) numbers were reduced. Moreover, white matter aberrations adjacent to the Aβ seeding were observed together with an overall decline in callosal myelination. This data indicate that the initial stages of plaque formation induce oligodendrocyte dysfunction, which might ultimately lead to myelin loss.

## Introduction

Alzheimer’s disease (AD) is a neurodegenerative disorder during which patients suffer from progressing cognitive impairment and memory loss. The most prominent neuropathological hallmarks are the extracellular aggregation of the amyloid-beta peptide (Aβ) in the form of amyloid plaques and intracellular neurofibrillary tangles. While for decades the deposition of Aβ plaques is implicated with the onset of AD, the abundance of Aβ-associated perturbations remains to be conclusively investigated.

Due to the stereotypical Aβ deposition pattern, by which Aβ plaques are mainly found in gray matter regions such as the cortex, thalamus, and hippocampus (Jucker and Walker, 2013), great focus is set on the investigation of Aβ-related gray matter disruptions. However, the corpus callosum (CC) atrophy seen in AD patients (Hampel et al., 1998) and myelin loss (Nasrabady et al., 2018), as well as the plaque-associated demyelination observed in an amyloid mouse model (Behrendt et al., 2013), raised awareness for the need of studying the involved pathomechanisms. Furthermore, a recent human single-cell transcriptomic study identified recurrent perturbations of myelination-associated genes in AD patients (Mathys et al., 2019), reinforcing the relevance of (de-) myelination in the context of AD.

Strikingly, a magnetic resonance imaging (MRI) study from the Dominantly Inherited Alzheimer’s Network (DIAN) including autosomal dominant AD mutation carriers assessed white matter abnormalities years before expected disease onset (Lee et al., 2016), indicating the loss of myelin integrity at an early disease stage. In the same study, said white matter aberrations occurred simultaneously with decreasing Aβ_42_ levels in the CSF of mutation carriers, implying a correlation between AD-associated demyelination and pre-symptomatic decline in CSF Aβ_42_ levels.

Therefore, we were wondering whether white matter integrity is already altered during the early stages of plaque formation. Through intracerebral injections of Aβ-containing brain extracts into pre-depositing AD transgenic mice, previous studies were able to robustly induce Aβ seeding, which represents the early phases of Aβ plaque formation (Kane et al., 2000; Meyer-Luehmann et al., 2006; Bachhuber et al., 2015; Ziegler-Waldkirch et al., 2018; Parhizkar et al., 2019; Katzmarski et al., 2020; Friesen et al., 2021; Ziegler-Waldkirch et al., 2022). Those seed-induced Aβ deposits, when injected into the hippocampus, could be encountered in the CC as well (Meyer-Luehmann et al., 2006), making it an intriguing model to study Aβ seed-induced alterations of the white matter. Utilizing this Aβ seeding model in pre-depositing 5xFAD mice (Friesen and Meyer-Luehmann, 2019), we report a pronounced astro- and microgliosis at the seeding site, accompanied by a reduction of oligodendrocyte progenitor cells (OPCs). Ultimately, Aβ seeding led to myelin anomalies adjacent to the seeding area and MRI scans further revealed CC demyelination. Our data thus provide one explanation for the reported early demyelination in AD patients by demonstrating that seed-induced diffuse Aβ can evoke the loss of white matter integrity before compact plaque formation.

## Materials and methods

### Animals

All animal experiments were carried out in accordance with the policies of the state of Baden-Württemberg under the license number G16/060. We used heterozygous 5xFAD transgenic mice co-expressing human APP^K670N/M671L (Sw) + I716V (Fl) + V717I^ ^(Lo)^ and PS1^M146L + L286V^ under the control of the neuron-specific Thy-1 promoter (Oakley et al., 2006). We backcrossed heterozygous 5xFAD to C57BL/6 mice to generate heterozygous 5xFAD mice and wildtype (WT) littermates. All mice had a C57BL/6 background. Only male mice were used in this study since female mice have a faster and earlier onset of Aβ plaque formation. Also, by using one gender, we wanted to minimize variability and reduce sample size. Animals were group-housed under specific pathogen-free conditions. Mice were kept under a 12-h light, 12-h dark cycle with food and water *ad libitum*.

### Preparation of brain homogenates for intracerebral injections

Mouse brain homogenates were derived from 10-month-old plaque-bearing heterozygous 5xFAD transgenic mice and age-matched non-transgenic littermates. Homogenates were obtained from the whole mouse brain. Brain tissue samples were fresh-frozen and stored at −80°C until use. Samples were homogenized in sterile phosphate-buffered saline (PBS) at 10% (w/v) and sonicated 3 × 5 s (30% amplitude, DigitalSonifier W-250D, Branson Ultrasonics). The crude brain homogenate was centrifuged for 5 min (at 3,000 *g*, 4°C) and the supernatant was stored at −80°C until use.

### Intracerebral stereotactic injections

The animals were anesthetized *via* intraperitoneal injection with a mixture of ketamine (100 mg/kg body weight) and xylazine (5 mg/kg body weight) in saline. For bilateral stereotactic injections of brain homogenates, a Hamilton syringe was placed into the hippocampus (AP −2.3 mm; L 2.0 mm; DV −2.0 mm) of 7-week-old male 5xFAD and WT mice as described previously (Bachhuber et al., 2015). The animals were injected either with 5xFAD transgenic brain homogenate or WT brain homogenate (2.5 μl per hemisphere at an injection speed of 1.25 μl/min) or were left uninjected as control animals. After each injection, the needle was kept in place for an additional 2 min before it was slowly withdrawn. The surgical site was cleaned with sterile saline and the incision was sutured. Mice were monitored until recovery from anesthesia and incubated for 5 or 10 weeks, respectively.

### Immunoblot analysis of injected brain homogenates

Samples were separated by 4–12% NuPAGE Bis-Tris mini gels utilizing NuPAGE LDS sample buffer, NuPAGE sample reducing agent, and NuPAGE MES SDS running buffer (Invitrogen). Proteins were transferred onto PVDF membranes (BioRad) and visualized using Clarity Western ECL Substrate (BioRad). To detect Aβ, the antibody 6E10 (mouse, 1:3,000, Covance) was used. The following peptides were used as standard and prepared (1:1 mixture) according to the manufacturer’s instructions: rPeptide Aβ1-40 HFIP (1 mg) (cat. no.: A-1153-2, lot no.: 2061240H) and rPeptide Aβ1-42 HFIP (1 mg), (cat. no.: A-1163-2, lot no.: 06021342H). ImageJ (National Institutes of Health freeware, version 1.52a) was used for densitometric analysis.

### Histology

Mice were transcardially perfused with 10 ml of ice-cold PBS followed by 10 ml of ice-cold 4% paraformaldehyde (PFA) in PBS (Roti-Histofix, Roth). Brains were isolated and post-fixed in 4% PFA for 24 h, followed by incubation in 30% sucrose (in PBS, pH 7.5) for a further 48 h. Frozen brains were cut into 25 μm thick coronal sections on a sliding microtome (SM2000R, Leica Biosystems, Wetzlar, Germany) and collected in 15% glycerol. Sections were blocked in 5% normal goat serum (NGS) in PBS with 0.5% Triton X-100. Afterward slices incubated overnight at 4°C with antibodies diluted in PBS containing 1% NGS against the following: anti-Aβ (mouse, 1:1,000, Covance, 6E10), anti-Iba1 (rabbit, 1:1,000, WAKO, 019-19741), anti-CD68 (rat, 1:500, BioRad, MCA1957), anti-GFAP (rabbit, 1:3,000, DAKO, Z033401-2), anti-C3 (rat, 1:200, abcam, ab11862), anti-PDGFRα (rabbit, 1:400, Cell Signaling, 3174), and anti-MBP (rabbit, 1:1000, abcam, ab40390). Corresponding secondary antibodies conjugated to Alexa 488 or 555 (1:1,000) were used. Sections were counterstained with DAPI (Sigma, D9542, 1:10,000) and mounted with a fluorescence mounting medium (DAKO, S3023).

### Assessment of Aβ and image analysis

Fluorescence images of brain slices were taken using a Zeiss fluorescent microscope (Axio Imager M2M). Confocal micrographs were acquired with either an Olympus Fluoview FV1000 or a Zeiss LSM880 AiryScan confocal microscope. For analysis, every 10th brain section approx. 1 mm anterior and posterior to the injection site were immunostained, with an interval of 250 μm per section. The definition of the CC was based on the mouse brain atlas (Franklin and Paxinos, 2019).

Total Aβ load was determined by calculating the relative areal fraction occupied with Aβ-positive staining (in %) in the CC using the image analysis software ImageJ (National Institutes of Health freeware, immunoblot analysis of injected brain homogenates). About four to seven animals per group and four to five sections per animal were analyzed.

Cell number was quantified by counting the number of positively labeled cells in the CC of the animals. This was mostly done in a semi-automated manner using custom-written macros. Since GFAP exclusively stains the processes of reactive astrocytes, the assessed cell number was substituted by the relative areal fraction occupied with the GFAP-positive signal (in %). To measure the thickness of the CC, six to seven animals per group and three to five sections per animal were analyzed. All analyses were conducted in a blinded manner.

### Gene expression analysis

We collected the CC tissue and stored it immediately at −80°C. Afterward, the tissue was homogenized in RLT lysis buffer by mechanical dissociation (RNeasy Mini Kit, Qiagen).

Then, RNA was isolated with the RNeasy Mini Kit (Qiagen) according to the manufacturer’s protocol. Reverse transcription and real-time PCR analysis were performed using the High-Capacity cDNA Reverse Transcription Kit and Gene Expression Master Mix reagents (Applied Biosystems) according to the manufacturer’s recommendations. qPCRs were analyzed with a LightCycler 480 (Roche). For gene expression analysis, we used the following TaqMan Gene Expression Assays: Actb (Mm01205647_g1), Apoe (Mm01307193_g1), C3 (Mm01232779_m1), Ccl5 (Mm01302427_m1), Cd109 (Mm00462151_m1), Cxcl10 (Mm099999072_m1), Emp1 (Mm00515678_m1), Gfap (Mm01253033_m1), Ggta1 (Mm01 333302_m1), and Serping1 (Mm00437835_m1).

### Electron microscopy

Mice were transcardially perfused with 20 ml of ice-cold PBS followed by 20 ml of ice-cold 4% PFA with 0.1% Glutaraldehyde in PBS. Brains were isolated and post-fixed in 4% PFA + 0.1% Glutaraldehyde for 24 h. After embedding the tissue in 5% agarose, 50 μm coronal sections of the CC were obtained using a vibratome (Leica VT1000 S).

Sections were post-fixed in 1% OsO_4_ in 0.1 mM phosphate buffer (PB) for 40 min. After 6 × 5 min washing in PB, slices were dehydrated in an ascending ethanol series (50%, 60%, 70% + 1% uranyl acetate, 80%, 90%, 96%, 100%) and treated with propylene oxide (2 × 10 min) followed by a 1:1 mixture of propylene oxide and durcupan (1 h) and ultimately with pure durcupan (overnight). To initialize durcupan polymerization and finalization of the embedding procedure, the tissue was placed in a 55°C oven for 48 h. Ultrathin 40 nm thick sections of the region of interest were cut utilizing an ultramicrotome (Leica UC6, Reichert-Jung) before transfer onto a copper slot grid for transmission electron microscopy (TEM) (Philips CM100). For g-ratio assessment of the axons, the axonal diameter and the corresponding diameter of axon + myelin sheath were measured using ImageJ. Subsequently, the diameter of the axon was divided by the entire fiber (g-ratio = diameter of the axon/diameter axon + myelin sheath). For each axon, two g-ratios were assessed (orthogonally to each other) and the mean was determined. For each animal, 75 to 204 axons were measured.

### Diffusion tensor imaging using magnetic resonance imaging

About 5 and 10 weeks post-intracerebral stereotactic injections, MRI scans were conducted. MRI experiments were performed while continuously monitoring body temperature and respiratory rate for maintenance of constant physiological levels (body temperature: 35.5 ± 1.5°C; respiratory rate: 50–60 breaths/min). For the morphological respiratory-gated T2-weighted and diffusion tensor imaging (DTI) acquisitions, anesthesia was induced with 4.0% and maintained with 1.5% isoflurane (Forene; Abbvie Deutschland GmbH & Co. KG, Wiesbaden, Germany), in 1.2 l oxygen/min. The total scanning time ranged from 45 to 50 min. After the experiment, animals were allowed to spontaneously recover.

### Data acquisition

All acquisitions were performed with a 7-T small horizontal bore animal scanner (Biospec 70/20, Bruker, Ettlingen, Germany), a mouse head adapted cryocoil (MRI CryoProbe, Bruker, Ettlingen, Germany), and ParaVision 6.0.1 (Bruker, Ettlingen, Germany). Before the data acquisition, a localized shimming procedure on a volume of interest placed inside the mouse brain was performed *via* field map and press waterline routines implemented in ParaVision 6.0.1 as described previously (Harsan et al., 2013).

High-resolution T2-weighted morphological images were acquired using a RARE sequence (TE/TR = 40 ms/4,000 ms), 24 slices of 0.3 mm thickness, sampling in an interlaced fashion, and 2 averages at a RARE factor of 4. An acquisition matrix of 256 × 196 and a FOV of 1.3 × 1 cm^2^ led to a spatial resolution of 0.051 × 0.051 × 0.3 mm^3^.

Structural connectivity was investigated based on DTI data. Acquisitions were performed using four shot DT-EPI (TE/TR = 47 ms/2,000 ms) sequence, with diffusion gradients applied along 30 non-collinear directions, a b_factor_ of 1,000 s/mm^2^ (Jones, 2004), diffusion gradient’s duration, and separation of 5 ms (δ) and 14 ms (Δ), respectively. A total of 12 brain slices of 0.6 mm thickness were obtained with a FOV of 1.3 cm × 1 cm and an acquisition matrix of 128 × 98 resulting in an image resolution of 0.101 × 0.101 mm^2^. As Jones (2004) showed, 30 unique sampling orientations are more than sufficient for a robust estimation of anisotropy and tensor orientation.

### Post-processing of diffusion tensor imaging data

Post-processing of the DTI data and fiber tracking with de-noising and un-ringing was performed as described previously (Hübner et al., 2017) using the in-house developed MATLAB-based DTI&FiberTool software package that is implemented in the imaging platform, NORA.^1^ The platform provides tools to manage, annotate, and post-process on cloud-based datasets. The global fiber tracking algorithm used in our study reconstructs highly accurate fiber bundles (Fillard et al., 2011) and has previously been validated for mouse and human DTI data (Harsan et al., 2013). The used method reconstructed all fiber bundles simultaneously for the whole brain without the requirement of defining seed or target regions. This approach offers resistance to the local imaging artifacts, avoiding the cumulative errors generally arising when sequentially integrating local fiber directions from predefined seed points. It also allows the reconstruction of a larger field of view when an ambiguous area had to be resolved. The reconstructed fibers are built with small line segments (particle) described by a spatial position and orientation. These segments are the basic building blocks of a fiber model, bounding together during the optimization procedure and forming longer chains that represent the individual fibers. Their orientation and number are adjusted simultaneously and the connections between segments are formed based on a probabilistic hopping that retains features of the probabilistic tracking algorithms (Reisert et al., 2011; Harsan et al., 2013). To generate fiber density (FD) maps, the number of tracts in each element of a grid was calculated from whole mouse brain fibers in a manner very similar to previously published methodology (Calamante et al., 2012). The method used the continuity information contained in the fibers reconstructed during the global tracking procedure to introduce subvoxel information based on supporting information from neighboring voxels. After the generation of the sufficient number of fibers passing a voxel at different spatial locations, their density was used as intravoxel information to construct the FD map. The directionality of the fibers was incorporated into the FD by assigning red/green/blue color to different spatial directions: red: mediolateral, green: dorsoventral, and blue: rostrocaudal orientation; thus generating highly resolved spatial histograms of diffusion orientations referred to as high-resolution fiber map.

### Statistical analysis

GraphPad Prism 6 was used for statistical analysis. Data sets were tested for normality (D’Agostino-Pearson Omnibus K2 normality test; significance level *p* = 0.05) before employing the appropriate parametric or non-parametric statistical comparison test. Applied statistical tests are depicted in the respective figure legends. Reported values represent the means ± SEM. To assess correlation, the Pearson correlation analysis was used. Significance level α was set to 0.05. *p < 0.05; ^**^p < 0.01; ^***^p < 0.001.

## Results

### Intracerebral injections provoke Aβ seeding in the corpus callosum of 5xFAD mice

**FIGURE 1:**
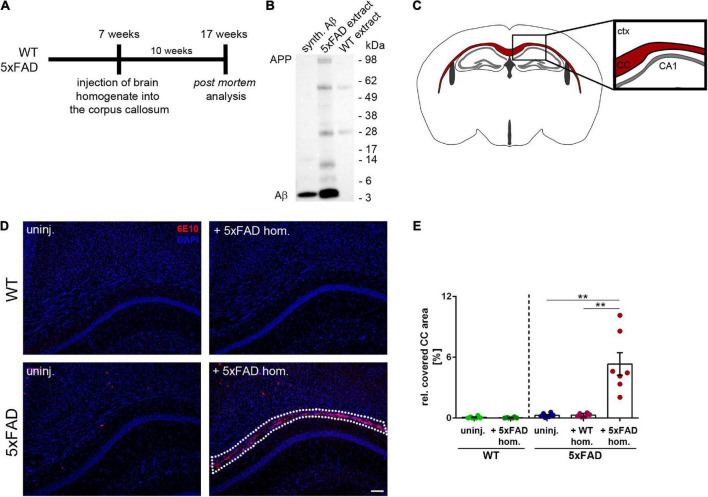
Intrahippocampal injections of amyloid-beta peptide (Aβ)-containing brain homogenate induces Aβ seeding in the corpus callosum (CC). **(A)** Timeline for the Aβ seeding experiments. **(B)** Representative immunoblot from whole brain extracts of aged 5xFAD and WT mice compared to synthetic (synth.) Aβ (mixture of synthetic Aβ1-40 and Aβ1-42). Immunoblot was probed with the 6E10 antibody. **(C)** Illustration of the region of interest (CC; indicated in red) used for subsequent image analysis. Abbreviations: Ctx, cortex; CA1, hippocampal CA1 region; CC, corpus callosum. **(D)** Fluorescence microscopy visualizing Aβ-material (6E10; red) of 4-month-old WT and 5xFAD mice, which were either uninjected (uninj.) or injected with Aβ-containing brain homogenate of an aged 5xFAD mouse (+5xFAD hom.). Dashed line indicates the Aβ seeding area. **(E)** Quantification of the Aβ load relative to the CC. *n* = 6–7 mice with *n* = 5 sections analyzed per animal. Each symbol represents data from one animal. Mean ± SEM, Kruskal–Wallis test followed by Dunn’s multiple comparison test, **p < 0.01. Scale bar 100 μm.

To examine the impact of seed-induced Aβ deposits on white matter integrity, we first performed intracranial injections of Aβ-containing brain homogenate from a depositing 5xFAD mouse into the hippocampus of young pre-depositing male 5xFAD mice and sacrificed the mice for *post-mortem* analysis after an incubation time of 10 weeks (Figure 1A). Immunoblotting of the 5xFAD brain extract that was used for intracerebral injections revealed monomeric, dimeric, trimeric, and larger oligomeric species (Figure 1B) with an Aβ concentration of 9.6 ng/μl in three representative 5xFAD homogenates. This value is consistent with those reported in previous studies (Meyer-Luehmann et al., 2006; Eisele et al., 2009). For the histological analysis, the CC was examined in coronal sections (Figure 1C).

While the 5xFAD mouse model exhibits numerous dense-core plaques in the cortex, thalamus, subiculum, and several other brain regions (Oakley et al., 2006), the number of plaques found in the CC of uninjected 5xFAD mice was low at the time of analysis (Figure 1D). However, in line with a previously published study (Meyer-Luehmann et al., 2006), we observed massive seed-induced Aβ deposits in the CC of 5xFAD mice that received injections of Aβ-containing brain homogenate (Figures 1D,E). In contrast, neither uninjected nor with WT homogenate injected 5xFAD mice developed Aβ seeding and only occasionally developed endogenous plaques in our region of interest. WT littermates, which were used as controls, remained also devoid of plaques, as previously reported (Kane et al., 2000; Meyer-Luehmann et al., 2006; Ziegler-Waldkirch et al., 2018). Quantification of the seeding area in the CC confirmed that only 5xFAD mice that were injected with Aβ-containing brain homogenate developed Aβ seeding, while all other groups of mice were devoid of seed-induced Aβ plaques (Figure 1E).

### Aβ seeding-induced gliosis in the white matter

**FIGURE 2:**
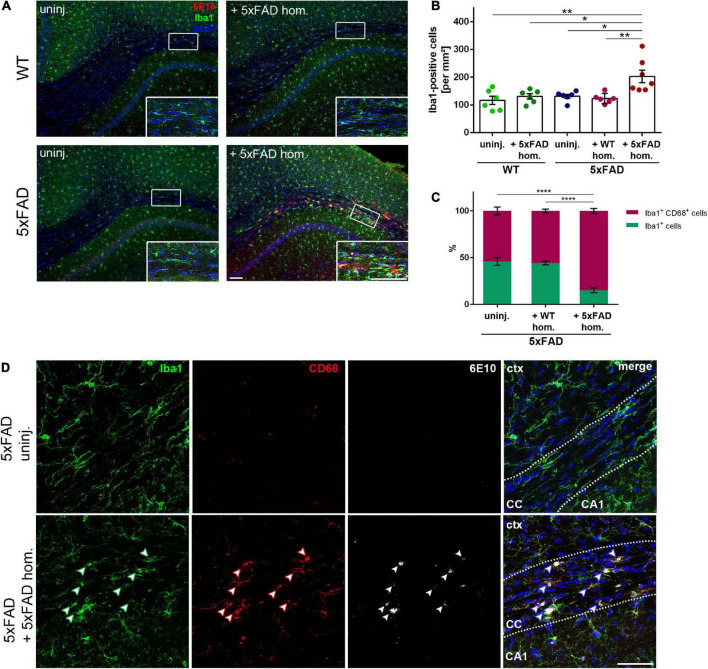
Enhanced microglia density and phagocytic activity in the corpus callosum (CC) of amyloid-beta peptide (Aβ)-seeded mice. **(A)** Immunofluorescent Iba1 (green) and 6E10 (red) staining of 4-month-old WT and 5xFAD mice, which were either uninjected or injected with 5xFAD brain homogenate. Scale bar for overview and inset 100 μm. **(B)** Respective quantification of Iba1-positive cells in the CC. *n* = 6–7 mice with *n* = 4–5 sections analyzed per animal. Each symbol represents data from one mouse. Mean ± SEM, Kruskal–Wallis test followed by Dunn’s multiple comparison test, *p < 0.05, **p < 0.01. **(C)** Quantification of the ratio between Iba1 single-positive and Iba1 and CD68 double-positive cells in the CC of 5xFAD animals, either uninjected or injected with either WT or 5xFAD homogenate. *n* = 4–5 mice with *n* = 5 sections analyzed per animal. Mean ± SEM, two-way ANOVA followed by Tukey’s multiple comparison test, ****p < 0.0001. **(D)** Confocal images of Iba1 (green), CD68 (red), and 6E10 (white) of 5xFAD mice, either not injected or injected with 5xFAD brain extract. Dashed line marks the CC. Arrowheads indicate microglial CD68 expression adjacent to Aβ deposits. Abbreviations: Ctx, cortex; CA1, hippocampal CA1 region; CC, corpus callosum. Scale bar 50 μm.

The involvement of glial cells in AD pathogenesis is an extensively studied topic, though several aspects of their contribution to the disease remain uncertain. Predominantly, microglia have gained a lot of attention, alternating between agonistic and antagonistic roles in the disease progression (Meyer-Luehmann and Prinz, 2015). Just recently, we could show that microglia can contribute to Aβ propagation into unaffected brain tissue (d’Errico et al., 2022). To determine the effect of seed-induced Aβ deposits on microglia, we performed double-immunofluorescence stainings with the microglia marker Iba1 together with 6E10 for Aβ. Interestingly, we found a significant increase of Iba1-positive cells adjacent to the seeding site, while in the non-seeded 5xFAD animals, the number of microglia did not differ compared to the WT animals (Figures 2A,B). When evaluating the Iba1 and CD68 co-immunoreactivity, the ratio of double-positive cells was significantly enhanced in Aβ-seeded mice (Figure 2C), indicating enhanced microglial phagocytic activity. Additionally, the microglial CD68 signal is frequently co-localized with Aβ seeding (Figure 2D).

**FIGURE 3:**
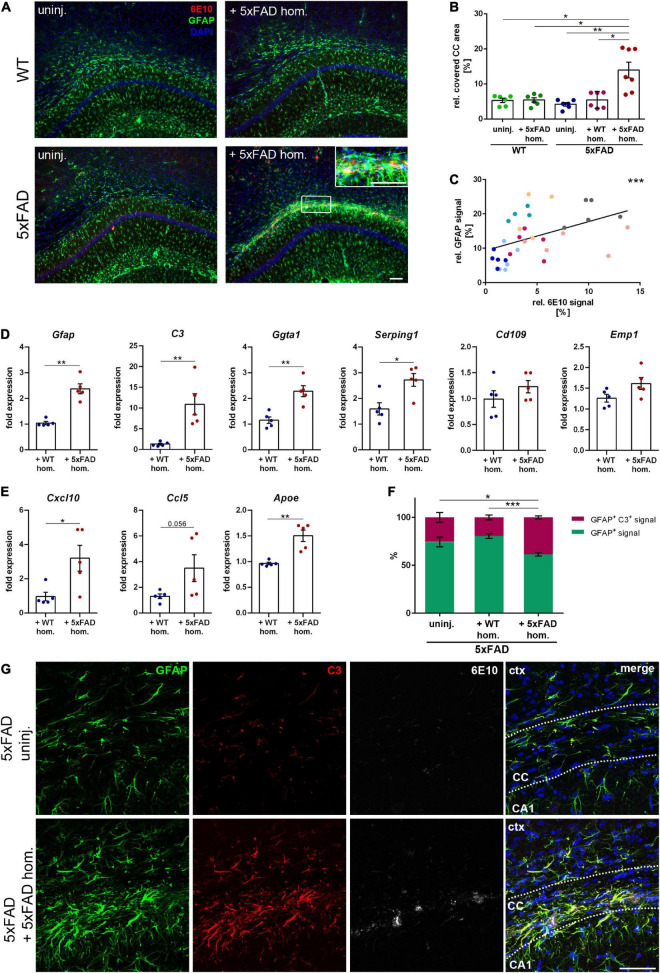
Reactive astrogliosis in the corpus callosum (CC) adjacent to the amyloid-beta peptide (Aβ) seeding. **(A)** Representative fluorescence micrographs of GFAP (green) and 6E10 (red) of 5xFAD brain homogenate-injected or uninjected 4-month-old WT and 5xFAD mice. Scale bar for overview and inset 100 μm. **(B)** Analysis of the GFAP signal relative to the CC area. *n* = 6–7 mice with *n* = 4–5 sections analyzed per animal. Each symbol represents data from one animal. Mean ± SEM, Kruskal–Wallis test followed by Dunn’s multiple comparison test, *p < 0.05, **p < 0.01. **(C)** Pearson correlation plots of the amount of Aβ seeding (%) relative to GFAP signal (%) in 5xFAD mice injected with 5xFAD homogenate. Each symbol represents data from one section and each color represents one mouse. *r* = 0.5752, ***p < 0.0005. **(D)** RT-qPCR of the astrocyte-associated genes *Gfap*, *C3*, *Ggta1, Serping1*, *Cd109*, and *Emp1* in 5xFAD mice injected with either WT (*n* = 5) or 5xFAD (*n* = 5) homogenate relative to 5xFAD uninjected mice (*n* = 5). Data were normalized to the housekeeping gene *Actb*. Mean ± SEM, unpaired non-parametric *t*-test (Mann–Whitney), *p < 0.05, **p < 0.01. **(E)** Gene expression of the chemokines *Cxcl10* and *Ccl5* as well as *Apoe* in 5xFAD animals with WT (*n* = 5) or 5xFAD (*n* = 5) homogenate relative to uninjected 5xFAD mice (*n* = 5). Data were normalized to *Actb*. Mean ± SEM, unpaired non-parametric *t*-test (Mann–Whitney), *p < 0.05, **p < 0.01. **(F)** Quantification of the ratio between GFAP signal and GFAP and C3 co-localizing signal in the CC of 5xFAD animals, either uninjected or injected with WT or 5xFAD homogenate. *n* = 4–6 mice with *n* = 4–5 sections analyzed per animal. Mean ± SEM, two-way ANOVA followed by Tukey’s multiple comparison test, *p < 0.05, ***p < 0.0005. **(G)** Confocal images of GFAP (green), C3 (red), and 6E10 (white) of 5xFAD mice, either not injected or injected with 5xFAD brain extract. Dashed line marks the CC. Abbreviations: Ctx, cortex; CA1, hippocampal CA1 region; CC, corpus callosum. Scale bar 50 μm.

Since it is well known that astrogliosis is associated with Aβ deposits in AD brains, we next assessed the response of reactive astrocytes to callosal Aβ seeding. In 5xFAD mice with seed-induced Aβ deposits, we observed a strong accumulation of GFAP-positive reactive astrocytes (Figures 3A,B). The detected astrogliosis seemed to be strongly associated with the seeding and even partially co-localized with the acquired Aβ signal (Figure 3A inset). Furthermore, the amount of GFAP-positive signal correlated significantly with the intensity of the seeding (Figure 3C), which was not the case for microglia (data not shown). To further characterize the reactive astrocytes responding to the Aβ seeding, we evaluated transcriptional levels of astrocyte-associated genes. Consistent with the immunofluorescence staining, gene expression of *Gfap* was elevated in Aβ-seeded animals, along with A1 astrocyte-associated genes *C3*, *Ggta1*, and *Serping1* (Figure 3D). Expression of A2 astrocyte-associated genes *Cd109* and *Emp1* was enhanced as well, albeit not significantly (Figure 3D). Moreover, transcriptional levels of the chemokines *Cxcl10* and *Ccl5*, as well as *Apoe*, were upregulated in the white matter of seeded mice (Figure 3E). Since the expression of C3 was drastically increased in Aβ-seeded mice and upregulated astrocytic C3 expression was previously observed in AD patients (Liddelow et al., 2017), as well as in APP transgenic mice (Lian et al., 2016), we determined C3 levels *via* immunofluorescence staining. Indeed, induced Aβ deposits in the CC led to significantly enhanced astrocytic C3 levels, evident by the pronounced co-localization and increased ratio of GFAP and C3 double-positive signal (Figures 3F,G). Taken together, as a response to the seed-induced Aβ material, pro-inflammatory cytokine expression was enhanced and microglia as well as astrocytes became activated.

### Decline in oligodendrocyte progenitor cell number and aberrant white matter myelination upon the development of Aβ deposits

**FIGURE 4:**
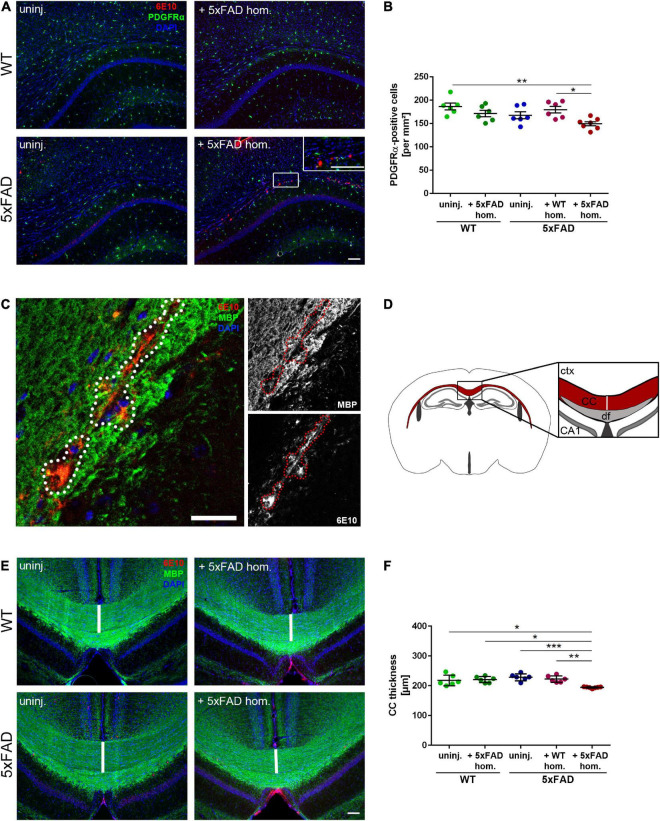
Reduced oligodendrocyte progenitor cell (OPC) number and disrupted white matter integrity in the corpus callosum (CC) exhibiting amyloid-beta peptide (Aβ) deposits. **(A)** Representative immunofluorescent staining of PDGFRα (green) and Aβ (red) of 4-month-old WT and 5xFAD mice, which were either uninjected or injected with 5xFAD brain homogenate. Scale bar for overview and inset 100 μm. **(B)** Respective analysis of PDGFRα-positive cells in the CC. *n* = 6–7 mice analyzing *n* = 4–5 sections per animal. Each symbol represents data from one mouse. Mean ± SEM, Kruskal–Wallis test followed by Dunn’s multiple comparison test, *p < 0.05, **p < 0.01. **(C)** Confocal microscopy of MBP (green) and Aβ (red) of 4-month-old 5xFAD mouse injected with 5xFAD brain homogenate. Dashed line indicates focal absence of MBP signal, which co-localizes with Aβ. Scale bar 30 μm. **(D)** Graphic representation of the region of interest (indicated as a white line) in the CC (red) used to quantify the CC thickness. Abbreviations: Ctx, cortex; CA1, hippocampal CA1 region; df, dorsal fornix; CC, corpus callosum. **(E)** Fluorescence microscopy visualizing MBP (green) and Aβ (red) of 4-month-old WT and 5xFAD mice, which were either uninjected or injected with 5xFAD brain homogenate. Scale bar 100 μm. **(F)** Respective quantification of the CC thickness. *n* = 6–7 mice with *n* = 3–5 sections analyzed per animal. Each symbol represents data from one animal. Mean ± SEM, Kruskal–Wallis test followed by Dunn’s multiple comparison test was used, comparing the Aβ seeded animals to the other 5xFAD groups. *p < 0.05, **p < 0.01, ***p < 0.0005.

According to a previous study, plaques are associated with focal demyelination and disrupt oligodendrocyte lineage dynamics (Behrendt et al., 2013). Therefore, we were wondering, whether our seed-induced Aβ deposits, which reflect an earlier stage of the dense core plaque formation, also lead to oligodendrocyte-related abnormalities. As a maker for OPCs, we opted for PDGFRα since PDGFRα-expressing cells were shown to generate myelinating oligodendrocytes in adult mice (Rivers et al., 2008). Interestingly, the number of PDGFRα-positive cells was significantly reduced in the CC of Aβ-seeded mice in comparison to all other control groups (Figures 4A,B). By myelin basic protein (MBP) immunolabeling, we subsequently wanted to assess whether seed-induced Aβ deposits perturbed the callosal myelination. Indeed, the MBP signal was absent in close proximity to the Aβ seeding area and revealed gaps in the form of black holes (Figure 4C). To determine the gross presence of myelin, we further evaluated the thickness of the CC (Figure 4D) and found a slight but significant reduction of CC thickness in the seeded animals (Figures 4E,F).

Finally, to evaluate myelin integrity in more detail, we performed a structural analysis of the white matter using TEM. For the classification of myelin anomalies, we utilized criteria as reported before (Peters et al., 2000; Thurnherr et al., 2006). EM images disclosed, while non-significant, a moderately higher ratio of myelin defects in the white matter of seeded 5xFAD mice, such as excess cytoplasm in the inner loop, and myelin compaction deficits or demyelinated axons (Supplementary Figures 1A,B). Additionally, the g-ratio was diminished in the white matter of seeded mice (Supplementary Figure 1C), indicating reduced axonal myelination in those animals. We hence recapitulate that seed-induced Aβ deposits in 5xFAD transgenic mice elicited diminished OPC levels concomitant with a reduction in callosal thickness and myelin aberrations in the white matter.

### Magnetic resonance imaging scans reveal Aβ seeding-associated demyelination in the corpus callosum

**FIGURE 5:**
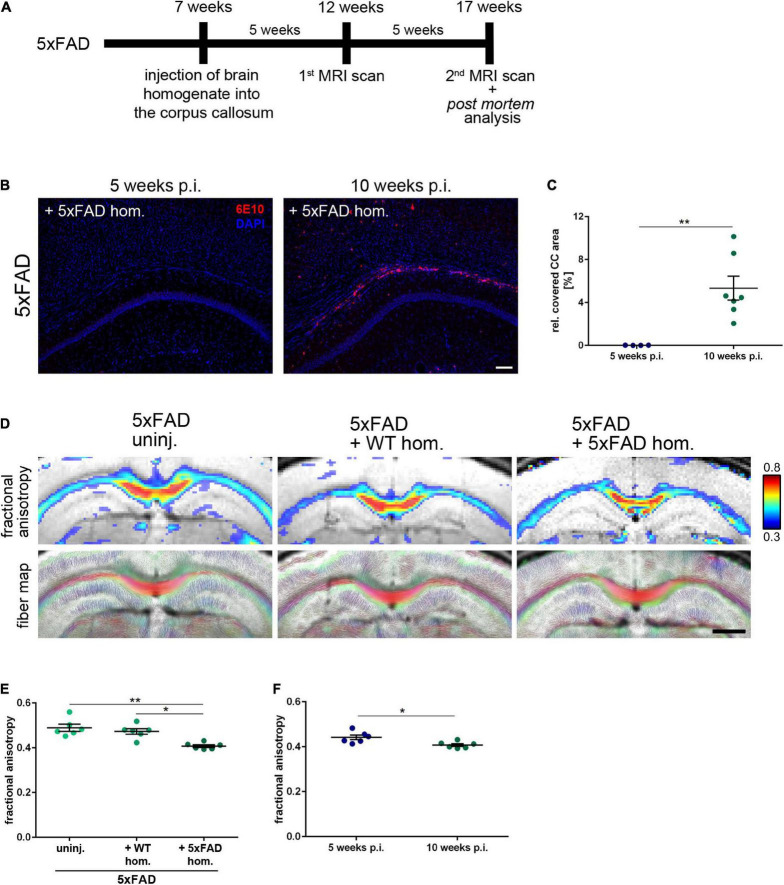
Seeding-associated demyelination in the corpus callosum (CC). **(A)** Timeline for the amyloid-beta peptide (Aβ) seeding experiments including the performed magnetic resonance imaging (MRI) scans 5 and 10 weeks post-injection (p.i.). **(B)** Representative fluorescent micrographs of the Aβ signal (red) of 4-month-old 5xFAD mice 5 and 10 weeks after the intrahippocampal inoculation of Aβ-containing brain homogenate. Scale bar 100 μm. **(C)** Analysis of the Aβ load relative to the CC. *n* = 4–7 mice with *n* = 5 sections analyzed per animal. Each symbol represents data from one animal. Mean ± SEM, unpaired non-parametric *t*-test (Mann–Whitney), **p < 0.01. **(D)** Representative visualization of the fiber density (FD) and high-resolution fiber map of 4-month-old 5xFAD mice 10 weeks p.i. with age-matched WT homogenate or 5xFAD homogenate and an uninjected control. Scale bar 1,000 μm. **(E)** Respective analysis of the callosal fractional anisotropy. *n* = 6 mice. Each symbol represents data from one mouse. Mean ± SEM, Kruskal–Wallis test followed by Dunn’s multiple comparison test, *p < 0.05, **p < 0.01. **(F)** Comparison of the fractional anisotropy from seeded 5xFAD mice 5 and 10 weeks p.i. with 5xFAD homogenate. *n* = 6 mice; Each symbol represents data from one animal. Mean ± SEM, unpaired non-parametric *t*-test (Mann–Whitney), *p < 0.05.

The observed focal demyelination and subtle decrease of callosal thickness pose the question of whether enhanced Aβ levels might also affect global white matter myelination. Thus, we conducted diffusion MRI scans of 5xFAD mice 5 and 10 weeks post-injection (p.i.) (Figure 5A) to visualize demyelination in the murine CC (Hübner et al., 2017). In contrast to the 10 weeks incubation time point, no Aβ-positive material could be found in the CC of 5xFAD mice at 5 weeks p.i. (Figures 5B,C). Accordingly, MRI scans of 5xFAD mice 5 weeks p.i. revealed no changes in fractional anisotropy, a read-out for myelin integrity, as well as in the fiber map when compared to the age-matched WT injected or uninjected 5xFAD animals (Supplementary Figures 2A,B). Intriguingly, and consistently with the aforementioned white matter anomalies, the measured fractional anisotropy, however, was significantly reduced, when those same animals were imaged 10 weeks p.i., that is, when Aβ seeding was evident (Figures 5D,E), indicating demyelination and white matter aberrations in those mice. Additionally, the fractional anisotropy was significantly diminished when compared to the pre-depositing time point (Figure 5F), leading to the conclusion that induced Aβ seeding in the CC of 5xFAD animals can interfere with white matter integrity.

## Discussion

In this study, we wanted to elucidate the relevance of early stages of Aβ deposition and aggregation on AD-associated disruption of myelin integrity. To model early Aβ aggregation stages, we took advantage of Aβ seeding and induced Aβ deposits in the CC, which triggered a prominent glial reaction and myelin aberration. Ultimately, MRI scans revealed white matter demyelination as a response to seed-induced Aβ deposits. The seeding model of Aβ pathology in mice offers a unique tool for studying the initial stage of Aβ plaque formation *in vivo* within a defined period of time (Friesen and Meyer-Luehmann, 2019). An additional benefit is the ability to study the direct effects of Aβ on the white matter without aging. Until now, the implications of altered white matter integrity in AD patients remain elusive. However, several mouse studies indicate the necessity of myelinogenesis for motor learning and memory formation (McKenzie et al., 2014; Pan et al., 2020). More importantly, cognitive deficits attributed to aging and observed in an AD mouse model were both reversible when myelination was enhanced (Wang et al., 2020; Chen et al., 2021), corroborating the relevance of myelin formation in an Alzheimer’s disease context, and suggesting a potential beneficial effect of pro-myelinating strategies for patients suffering from AD. Although human studies would be indispensable to evaluate the downstream effects of demyelination in AD, the present study reiterates the harmful consequences of Aβ accumulation on myelin integrity already at an early disease stage.

To our surprise, we detected fewer OPCs as a reaction to Aβ seeding, seemingly opposing previous findings which observed enhanced myelin renewal (Chen et al., 2021) as well as increased OPC levels in an amyloid mouse model (Behrendt et al., 2013). However, this reported that pronounced OPC proliferation was always accompanied by loss of white matter integrity suggesting similar mechanisms involved in the upregulation of OPC proliferation seen after demyelinating injuries (Franklin and Ffrench-Constant, 2008). Since our seeding model resembles the early stages of Aβ plaque deposition, we hypothesize that the Aβ seed-induced reduction in OPCs reflects the early stages of AD-associated alteration in oligodendrocyte dynamics. Although further examination of our seeding model would be essential to solidify our hypothesis, our data so far indicate myelin aberrations as a consequence of seed-induced Aβ deposits.

We detected a moderate micro- and even stronger astrogliosis in the CC of seeded 5xFAD mice. In addition, microglial CD68 expression was more pronounced in close proximity to the Aβ material, indicating an enhanced microglial phagocytic activity. Microglia play an essential role in myelinogenesis and OPC homeostasis (Hagemeyer et al., 2017). During postnatal white matter development, an amoeboid microglia population in the CC regulates myelinogenesis *via* clearance of excess myelin and phagocytosis of both OPCs (Nemes-Baran et al., 2020) and newly formed oligodendrocytes (Li et al., 2019). Further transcriptomic characterization of this white matter-associated microglia subset revealed a unique signature (Li et al., 2019), resembling AD-associated microglia (Keren-Shaul et al., 2017). Accordingly, we presume that Aβ seed-induced activation of phagocytic microglia might falsely trigger the pruning of oligodendrocyte lineage cells, culminating in myelin defects. This would go in line with the observed absence of cuprizone-mediated demyelination and loss of oligodendrocytes in microglia-depleted mice (Marzan et al., 2021). Additional indication for the activation of glial cells upon callosal Aβ seeding is the expression of pro-inflammatory cytokines. It is however, important to mention that the experimental setup does not allow to distinguish the origin of chemokine overexpression, that is, the cells which expressed these genes.

The severe accumulation of astrocytes and microglia adjacent to the Aβ seeding area accompanied by enhanced levels of pro-inflammatory cytokines and reduced OPC levels led us to speculate about the reasons for this “tri-glial imbalance.” It is well known that activated microglia can trigger the reactive A1 astrocyte subtype, which in turn induces the apoptosis of mature oligodendrocytes and impedes the proliferation and maturation of OPCs (Liddelow et al., 2017). This mechanism was later fortified by a study on chemotherapy-associated cognitive decline. Upon treatment with a chemotherapeutic agent, mice exhibited activated microglia as well as astrocytes, ultimately leading to myelin degeneration and cognitive deficits (Gibson et al., 2019). In immunofluorescence stainings of C3, an A1 astrocyte-associated marker, we observed an elevated ratio of C3-positive astrocytes close to the seed-induced Aβ deposits. Concordantly, transcriptional levels of *C3*, as well as the other A1 astrocyte-associated genes *Serping1* and *Ggta1*, were enhanced in the CC of seeded animals, underpinning our speculation. In accordance with the likewise, though not significant, increased expression of A2 astrocyte-associated genes, it is important to emphazise that functional readouts and *in vivo* approaches are vital to evaluate astrocytic behavior more reliably (Escartin et al., 2021) and would thereby be indispensable to unravel the exact mechanisms and the culprit(s) for the Aβ seeding-induced demyelination.

By performing electron microscopy, we saw structural aberrations in the callosal myelin sheaths of seeded 5xFAD mice. Along the same line, we observed focal demyelination in the presence of Aβ seeding in confocal images. Interestingly, *in vitro* experiments have shown that rat oligodendrocytes treated with Aβ exhibited stalled myelin sheath formation and diminished oligodendrocyte survival (Horiuchi et al., 2012). Furthermore, Aβ plaques were linked to focal myelin loss in both human and murine tissue (Mitew et al., 2010; Behrendt et al., 2013). It is however, important to note that in our study, diffuse Aβ material can evoke focal myelin degeneration before dense-core plaque formation. On a more macroscopic level, Aβ seeding led to significant callosal atrophy and MRI scans confirmed disturbed myelin integrity of the white matter, corroborating our data. Remarkably, these white matter aberrations were not detectable in the first scan 5 weeks p.i., when Aβ seeding was still absent, indicating that the white matter aberrations are linked to Aβ seeding.

In conclusion, this study demonstrates that seed-induced Aβ deposits in the CC of 5xFAD mice provoke pronounced reactive astrogliosis and increased numbers of phagocytic microglia, whereas the OPC levels were reduced. As a response to the Aβ seeding, focal demyelination adjacent to the Aβ material and overall corruption of myelin integrity in the white matter was visible. More efforts will be crucial to comprehend the impact of Aβ-associated white matter abnormalities and demyelination as well as to evaluate pro-myelinating approaches to combat myelin loss. Still, our data provides valuable insights into the glial response during the early phase of Aβ pathogenesis in the white matter.

## Data availability statement

The original contributions presented in the study are included in the article/Supplementary material, further inquiries can be directed to the corresponding author.

## Ethics statement

The animal study was reviewed and approved by the State of Baden-Württemberg.

## Author contributions

VA and MM-L conceived and planned the experiments, discussed the results, and wrote the manuscript. VA contributed to all aspects of the experiments and data analysis. VA, SZ-W, MF, DE, DL, and AH performed the experiments. DL and OK performed electron microscopy experiments. WR and DvE provided expertise in diffusion tensor imaging and magnetic resonance imaging. WR performed the MRI scans and data analysis. MM-L supervised the project and coordinated the study. All authors read, edited, and approved the manuscript.

## Footnotes

1
www.nora-imaging.org

## Supplementary material

Supplementary Figure 1 Myelin aberrations and reduced g-ratio in the corpus callosum of Aβ-seeded 5xFAD mice. **(A)** Electron microscopy of the corpus callosum from 4-month-old WT and 5xFAD mice, which were either uninjected or injected with either age-matched WT or 5xFAD brain homogenate. Examples for myelin alterations such as excess cytoplasm in the inner loop (arrow), demyelinated axons (arrowhead) and myelin compaction deficits (asterisk) are indicated, respectively. Scale bar 1 μm. **(B)** Quantification of white matter integrity, by assessing the ratio of intact and aberrant myelin (in percent). *n* = 2 mice. Mean ± SEM. Kruskal–Wallis test followed by Dunn’s multiple comparison test. Changes visible in the Aβ seeded 5xFAD group were not significant compared to the other groups. **(C)** g-ratio analysis of the electron micrographs. *n* = 2 mice with *n* = 184–329 measured axons per animal. Mean ± SEM. Each symbol represents data from one axon. Kruskal–Wallis test followed by Dunn’s multiple comparison test was utilized, comparing the Aβ seeded animals to the others groups. ^****^*p* < 0.0001.

Supplementary Figure 2 No evident changes in white matter myelination when Aβ seeding is still absent. **(A)** Visualization of the fiber density and high-resolution fiber map of 5xFAD mice 5 weeks post-injection of age-matched WT homogenate (+WT hom.) or 5xFAD homogenate and an uninjected control. Scale bar 1000 μm. **(B)** Respective analysis of the callosal fractional anisotropy. *n* = 6 mice. Mean ± SEM. Each symbol represents data from one animal. Kruskal–Wallis test followed by Dunn’s multiple comparison test.
