# Supplementary figures and images for: Seed-induced Aβ deposits in the corpus callosum disrupt white matter integrity in a mouse model of Alzheimer’s disease
Source: Front Cell Neurosci. 2022 Aug 8;16:862918. doi: 10.3389/fncel.2022.862918 (PMC9393256; doi:10.3389/fncel.2022.862918)

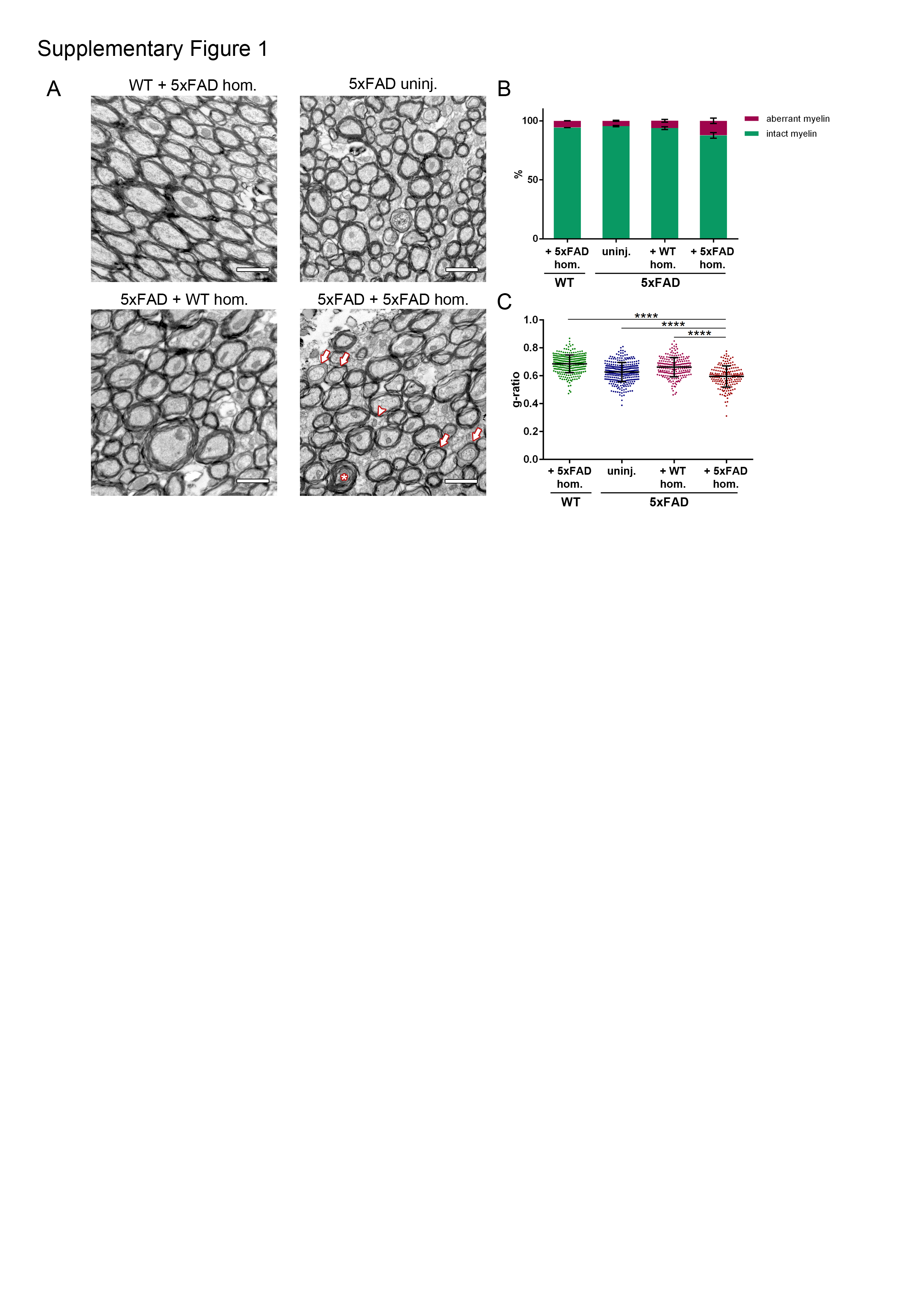

Supplement: Supplementary Figure 1 — Myelin aberrations and reduced g-ratio in the corpus callosum of Aβ-seeded 5xFAD mice. (A) Electron microscopy of the corpus callosum from 4-month-old WT and 5xFAD mice, which were either uninjected or injected with either age-matched WT or 5xFAD brain homogenate. Examples for myelin alterations such as excess cytoplasm in the inner loop (arrow), demyelinated axons (arrowhead) and myelin compaction deficits (asterisk) are indicated, respectively. Scale bar 1 μm. (B) Quantification of white matter integrity, by assessing the ratio of intact and aberrant myelin (in percent). n = 2 mice. Mean ± SEM. Kruskal–Wallis test followed by Dunn’s multiple comparison test. Changes visible in the Aβ seeded 5xFAD group were not significant compared to the other groups. (C) g-ratio analysis of the electron micrographs. n = 2 mice with n = 184–329 measured axons per animal. Mean ± SEM. Each symbol represents data from one axon. Kruskal–Wallis test followed by Dunn’s multiple comparison test was utilized, comparing the Aβ seeded animals to the others groups. ****p < 0.0001. [file Image_1.TIF]

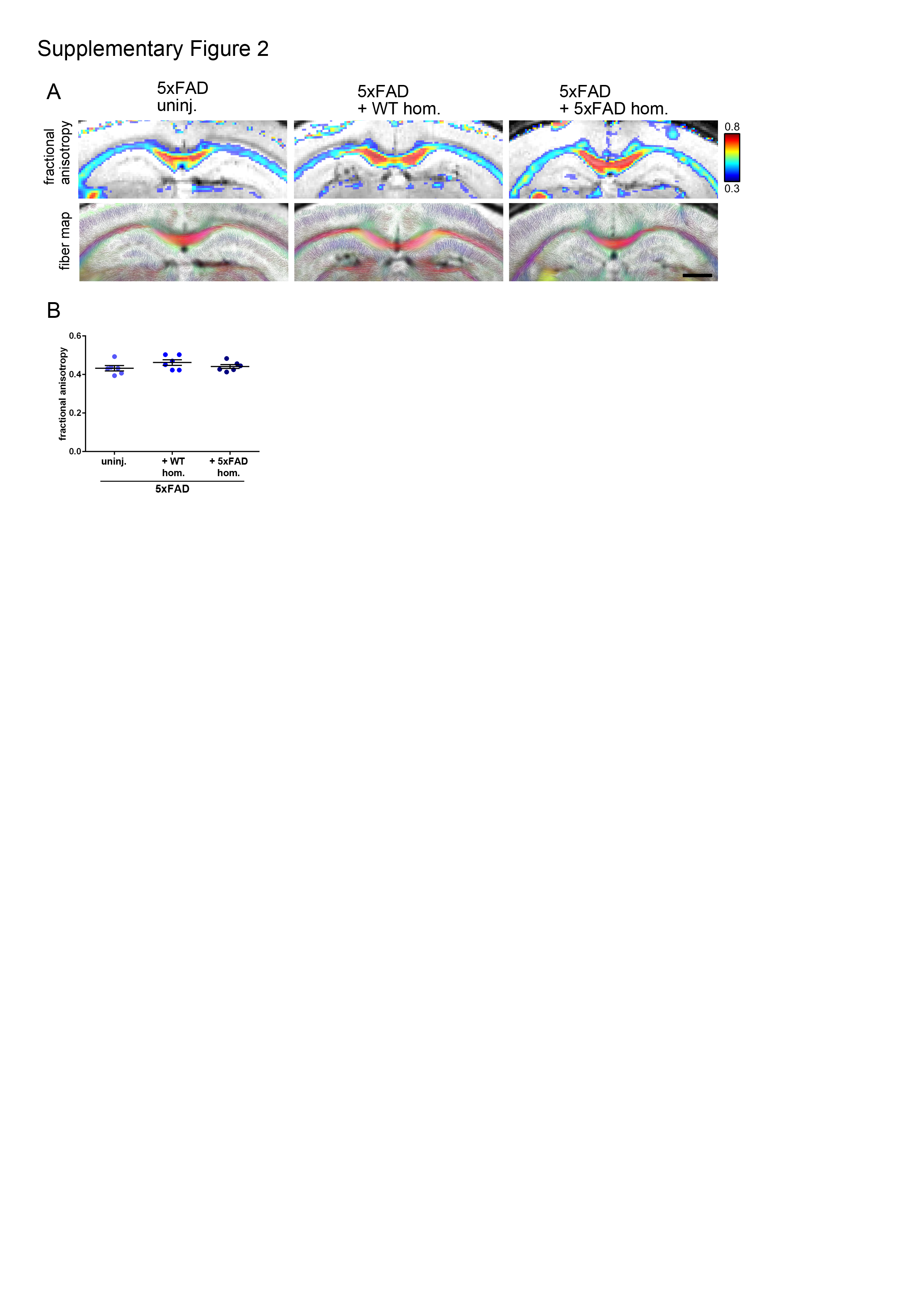

Supplement: Supplementary Figure 2 — No evident changes in white matter myelination when Aβ seeding is still absent. (A) Visualization of the fiber density and high-resolution fiber map of 5xFAD mice 5 weeks post-injection of age-matched WT homogenate (+WT hom.) or 5xFAD homogenate and an uninjected control. Scale bar 1000 μm. (B) Respective analysis of the callosal fractional anisotropy. n = 6 mice. Mean ± SEM. Each symbol represents data from one animal. Kruskal–Wallis test followed by Dunn’s multiple comparison test. [file Image_2.TIF]
